# Safety and efficacy of first-in-man intrathecal injection of human astrocytes (AstroRx®) in ALS patients: phase I/IIa clinical trial results

**DOI:** 10.1186/s12967-023-03903-3

**Published:** 2023-02-14

**Authors:** Marc Gotkine, Yoseph Caraco, Yossef Lerner, Simcha Blotnick, Maor Wanounou, Shalom Guy Slutsky, Judith Chebath, Graciela Kuperstein, Elena Estrin, Tamir Ben-Hur, Arik Hasson, Kfir Molakandov, Tehila Sonnenfeld, Yafit Stark, Ariel Revel, Michel Revel, Michal Izrael

**Affiliations:** 1 Department of Neurology, The Agnes Ginges Center for Human Neurogenetics, Hadassah-Hebrew University Medical Center, Jerusalem, Israel; 2 Neurodegenerative Diseases Department, Kadimastem Ltd, Pinchas Sapir 7, Weizmann Science Park, Ness-Ziona, Israel; 3 Hadassah Clinical Research Center (HCRC), Hadassah-Hebrew University Medical Center, Jerusalem, Israel; 4 Department of Molecular Genetics, Weizmann Institute of Science, 76100 Rehovot, Israel

**Keywords:** Cell therapy, ALS, Clinical trial, Astrocytes, Intrathecal injection

## Abstract

**Background:**

Malfunction of astrocytes is implicated as one of the pathological factors of ALS. Thus, intrathecal injection of healthy astrocytes in ALS can potentially compensate for the diseased astrocytes. AstroRx® is an allogeneic cell-based product, composed of healthy and functional human astrocytes derived from embryonic stem cells. AstroRx® was shown to clear excessive glutamate, reduce oxidative stress, secrete various neuroprotective factors, and act as an immunomodulator. Intrathecal injection of AstroRx® to animal models of ALS slowed disease progression and extended survival. Here we report the result of a first-in-human clinical study evaluating intrathecal injection of AstroRx® in ALS patients.

**Methods:**

We conducted a phase I/IIa, open-label, dose-escalating clinical trial to evaluate the safety, tolerability, and therapeutic effects of intrathecal injection of AstroRx® in patients with ALS. Five patients were injected intrathecally with a single dose of 100 × 10^6^ AstroRx® cells and 5 patients with 250 × 10^6^ cells (low and high dose, respectively). Safety and efficacy assessments were recorded for 3 months pre-treatment (run-in period) and 12 months post-treatment (follow-up period).

**Results:**

A single administration of AstroRx® at either low or high doses was safe and well tolerated. No adverse events (AEs) related to AstroRx® itself were reported. Transient AEs related to the Intrathecal (IT) procedure were all mild to moderate. The study demonstrated a clinically meaningful effect that was maintained over the first 3 months after treatment, as measured by the pre-post slope change in ALSFRS-R. In the 100 × 10^6^ AstroRx® arm, the ALSFRS-R rate of deterioration was attenuated from − 0.88/month pre-treatment to − 0.30/month in the first 3 months post-treatment (p = 0.039). In the 250 × 10^6^ AstroRx® arm, the ALSFRS-R slope decreased from − 1.43/month to − 0.78/month (p = 0.0023). The effect was even more profound in a rapid progressor subgroup of 5 patients. No statistically significant change was measured in muscle strength using hand-held dynamometry and slow vital capacity continued to deteriorate during the study.

**Conclusions:**

Overall, these findings suggest that a single IT administration of AstroRx® to ALS patients at a dose of 100 × 10^6^ or 250 × 10^6^ cells is safe. A signal of beneficial clinical effect was observed for the first 3 months following cell injection. These results support further investigation of repeated intrathecal administrations of AstroRx®, e.g., every 3 months.

*Trial Registration:* NCT03482050.

**Supplementary Information:**

The online version contains supplementary material available at 10.1186/s12967-023-03903-3.

## Background

Amyotrophic lateral sclerosis (ALS) is characterized by the loss of both upper and lower motor neurons (MNs). The symptoms include progressive paralysis of MN target muscles. The disease is incurable, and fatal within 3–5 years of first symptoms, usually due to respiratory failure when the diaphragm is affected [1]. The three FDA-approved drugs for the treatment of ALS, riluzole, edaravone, and the recently approved drug Relivrio™ (a combination of sodium phenylbutyrate/taurursodiol) have a modest effect on survival and disease progression [2–6], thus there is an urgent unmet need for therapies that can further delay the pathogenic process.

The pathological mechanisms of ALS are still not well understood and the proposed mechanisms include inflammation, oxidative stress, glutamate cytotoxicity, and protein aggregation. Although Motor Neurons (MNs) are the main affected cells in the disease, a growing body of evidence suggests the involvement of astrocytes in the pathogenesis of ALS in a non-cell-autonomous pathway [7, 8]. In healthy conditions, astrocytes support neurons in various ways. Astrocytes regulate the concentration of neurotransmitters and ions, supply a variety of metabolites and energy, regulate osmolarity, modulate synaptic activity, secrete neurotrophic and neuroprotective factors, promote neurogenesis [9, 10] and remyelination [11], and play a role in immunomodulation [12]. The contribution of astrocytes to the pathology of ALS is probably a combination of loss of homeostatic functions and/or gain of toxic functions [8, 13–15]. Recent studies also provide evidence for the beneficial role that astrocytes play in protecting MNs in ALS by reducing TDP-43 aggregates and secretion of neuroprotective factors [16–19]. Interestingly, correction of a pathogenic germline mutation in astrocytes alone slowed down MN degeneration [20]. Comprehensive preclinical studies demonstrated that transplantation of glial-precursor-cells that were generated from iPSCs, or embryonic-stem-cells (ESC), had the potential to delay disease onset and ameliorate clinical symptoms in rodent models of ALS disease [21–23] and shown to be safe [19]. Thus, transplantation of healthy astrocytes into the CNS of ALS patients could potentially compensate for malfunctioning endogenous astrocytes and attenuate the progression of the disease.

Pluripotent human embryonic stem cells are an excellent source for regenerative therapies as they can be produced in high quantities and can differentiate into most cell types of the body, including astrocytes [24], human astrocytes derived from clinical-grade embryonic stem cells demonstrated activities of functional healthy astrocytes, including glutamate uptake, secretion of various neurotrophic factors (e.g. GDNF, BDNF, TIMP-1, TIMP-2, and Midkine), promotion of axon outgrowth, immunomodulation and protection of MNs from oxidative stress [19]. Intrathecal injections of AstroRx® into transgenic hSOD1^G93A^ mice and rats significantly delayed disease onset and improved motor performance, as compared to control animals. A nine-month safety study in immunodeficient mice demonstrated the safety of AstroRx® treatment, as well as the biodistribution and survival of the cells upon intrathecal administration [19].

Here we report on the results of phase I/IIa, open-label, dose-escalating clinical study to evaluate the safety, tolerability, and therapeutic effects of intrathecal injection of AstroRx® cells in patients with amyotrophic lateral sclerosis.

## Methods

### Standard protocol approvals, registrations, and patient consent.

The study protocols were approved by the Israeli Ministry of Health (IMOH), and the institutional review board of Hadassah Medical Center in Jerusalem, Israel. All patients signed informed consent documents before the screening.

### Study objectives

The study aimed to evaluate the safety, tolerability, and therapeutic effects (preliminary efficacy) of a single intrathecal injection of low and high doses of AstroRx®, as a treatment for patients with ALS.

### Patients' selection criteria

Eligible participants were aged 18–70 years with a diagnosis of probable or definite ALS by revised El Escorial Criteria, within two years of diagnosis. The ALSFRS-R score was ≥ 30, and slow vital capacity (SVC) was ≥ 70% of the predicted normal value for height, age, and sex. Participants were either not receiving riluzole and/or edaravone or were on a stable dose for ≥ 30 days. Potential patients were excluded for the following reasons: past infection or a positive test for HBV, HCV, or HIV, need for respiratory support, renal failure, impaired hepatic function, Body Mass Index (BMI) of < 18.5 or > 30, significant cardiac disease, diabetes, autoimmune diseases, chronic severe infection, malignant disease or any other disease or condition that may risk the patient or interfere with the ability to interpret the study results. A full list of inclusion and exclusion criteria is shown in Additional file 1.

### Study outline

**Fig. 1:**
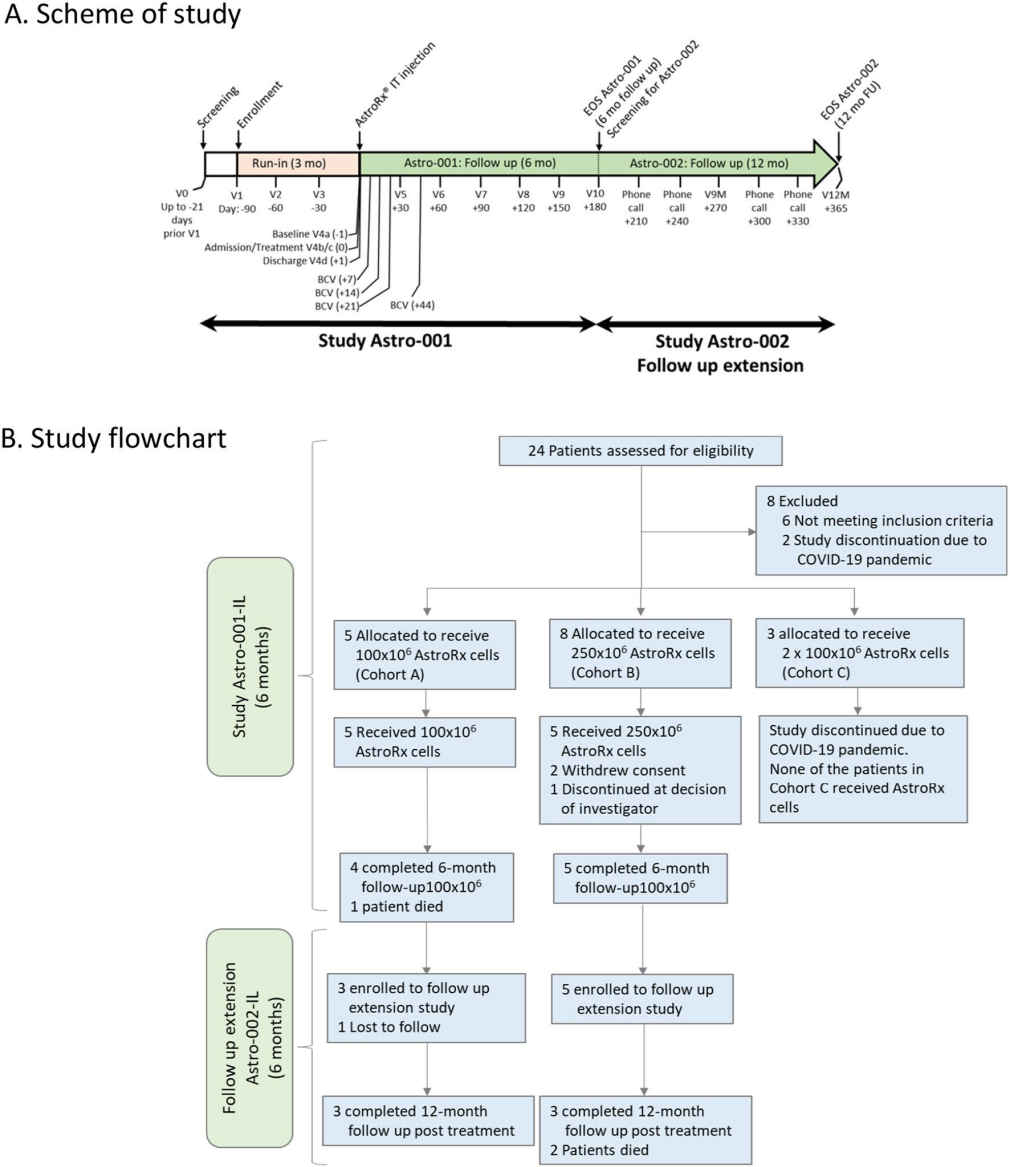
Phase 1/2a study design and study flowchart.** A** Visit 0 (V0) - screening visit, visit 1 (V1) till visit 4 (V4) presents about 3 months run-in period (pre-treatment), AstroRx injection was performed on V4. V4 till visit V10 is the 6 months follow up time under ASTRO-001 study and additional 6 months follow up was performed under study ASTRO-002 on V10-V12 and by phone call.** B** Study flow chart of patient allocation, treatment doses of ASTRO-001 and ASTRO-002.* V* Visit,* mo* Month,* BCV* Blood Count Visit,* EOS* End of Study

A diagram of the study design is shown in Fig. 1a. The study was conducted under 2 sequential clinical protocols, the interventional protocol Astro-001 and its extension, the non-interventional protocol, Astro-002.

Study Astro-001: following enrollment, the patients were monitored monthly during a run-in period of about 3 months to determine the progression rate of their ALS disease. Following the run-in period, patients were administered with 100 × 10^6^ AstroRx® cells (Group A, *n* = 5 patients) and 250 × 10^6^ cells (Group B, *n* = 5 patients) by a standard LP procedure. The immunosuppressive drug, Mycophenolate Mofetil (MMF) at 1 gr b.i.d. was given 2 days before the intrathecal cell injection and continued for an additional month (total of 32 days). Patients underwent weekly complete blood count (CBC) during the month of MMF treatment, and twice monthly following MMF cessation, to check for leukopenia.

After intrathecal AstroRx® injection, the patients were monitored monthly during a follow-up of 6 months. Upon completion of study Astro-001 (at a 6-month follow-up), participants were offered enrolment in the extension study Astro-002.

Under the study Astro-002, each patient was followed up monthly for an additional 6 months by either on-site visits or phone calls. The outcome measures were similar to study Astro-001. Safety data were monitored by the study investigators, medical monitor, and the Data and Safety Monitoring board (DSMB), which was independent of the study and sponsor.

The initial study design consisted of two additional arms of repeated doses of 100 × 10^6^ cells and 250 × 10^6^ separated by an interval of 60 days. However, due to the COVID-19 pandemic restrictions and the perception of the potential risks it posed to people with ALS, we adopted the recommendation of the independent DSMB and did not treat these 2 cohorts.

### AstroRx® Cell manufacturing

The clinical-grade AstroRx® cell product derived from human embryonic stem cells was manufactured under cGMP conditions in Kadimastem's GMP facility using standard operating procedures. AstroRx® cells were freshly prepared, harvested, and formulated in PlasmaLyte to reach a volume of 5 ml with 100 × 10^6^ or 250 × 10^6^ AstroRx® cells. The formulated drug product was uploaded into a 10 ml syringe and transported from the manufacturing facility to the clinical site in a validated shipping system at a controlled temperature of 2–8 °C and administered to the patient within 24 h from formulation. Validated safety quality control tests were performed before the release of each formulated AstroRx cell product before delivery to the clinical site, including sterility, mycoplasma, endotoxin, and Gram or HBL test performed by external qualified certified GLP laboratory (Hylabs laboratories, Israel). The viability and cell concentration were determined using an automated cell counter Nucleocounter (NC-200™ Chemometec®). The identity of AstroRx cell product was assessed by flow cytometry using the following antibodies: anti-GLAST (Miltenibiotec, 1:100), anti- CD44 (BD Pharmingen, 1:50), and anti-GFAP (Miltenibiotec, 1:50). Antibodies against SSEA-4 and EPCAM (both from Biolegend) were used for the detection of any pluripotent marker impurities. The Flow cytometer FACS Canto II (BD) operated with FACSDIVA software (BD) was used for the analysis. To assess AstroRx® potency *in-vitro*, AstroRx® cells secretion of Midkine and TIMP-1 was determined by ELISA using Human TIMP-1 Quantikine ELISA Kit (R&D systems) and Human Midkine ELISA Kit (Abcam). The optical density was read using the iMark Microplate reader (Bio-Rad Laboratories). A certificate of analysis was generated and approved by the quality assurance department to ensure that each released product met the release criteria before it was delivered to the clinical site for Intrathecal injection.

### Measurement outcomes

The primary objective of this study was to assess the safety and tolerability of AstroRx® in patients with ALS. Safety laboratory assessments were performed, and adverse events (AEs) were recorded at each visit. In addition, CNS imaging by MRI and CT scans was performed around 1 month before treatment, 1 month after treatment (CT), and 6 months after treatment (MRI).

The second objective was to evaluate the efficacy of intrathecal injection of AstroRx® in ALS. For this aim, data on ALSFRS-R, predicted slow vital capacity (SVC), hand-held dynamometry (HHD), and grip strength (using JAMAR plus) were collected at all pre-treatment and post-treatment on-site visits. ALSFRS-R score was also performed during home and phone visits. All tests were performed by trained evaluators who were certified by the Outcomes and Monitoring Center for the Northeast ALS Consortium. In addition, levels of the serum biomarker creatinine, creatine kinase, and neurofilament light chain (Nfl) were assessed in selected visits before and after treatment (Additional file 1).

### Serum neurofilament analysis

Serum samples for the analysis of Nfl as a biomarker for ALS progression were collected. In study Astro-001-IL, samples were collected on Visit 2 (day −60), Visit 3 (day −30), Visit 4a (day −1), Visit 4d (day + 1), Visit 5 (day + 30), Visit 7 (day + 90) and Visit EOS (day + 180). In the extension study Astro-002-IL, samples were collected at the Screening Visit (day + 180; EoS Visit in protocol Astro-001-IL), Visit 9 M (day + 270), and EOS Visit (day + 365). The measurement of the concentration of the biomarker in serum was performed using the validated SIOMA (Single Molecule Array) method by Quanterix (US).

### Statistical analysis

The primary trial outcome was safety, assessed for the incidence of treatment-emergent AEs (TEAEs) and serious AEs (SAEs), laboratory abnormalities, vital signs, ECGs, physical examinations, and CNS imaging. Medical history and AEs were coded using the Medical Dictionary for Regulatory Activities (MedDRA version 21). The Intent to Treat (ITT) population of the study included all subjects who were eligible to be enrolled to receive any study treatment. The Safety Analysis Set included a subset of the ITT set who had undergone intrathecal injection of AstroRx®.

The efficacy analyses were performed on the Modified ITT (mITT) population, which included a subset of the ITT that had undergone intrathecal injection of AstroRx® and had at least one post-baseline efficacy assessment. The baseline was defined for each subject as the last available, valid, non-missing assessment before the first study treatment administration. Efficacy endpoints were analyzed for the change between pre-treatment (run-in) period and post-treatment period. The nominal α level was 2-sided using α = 0.05. Since the study was an open-label exploratory study, no formal correction for type I error due to multiplicity was performed.

The Slope Analysis compared the slope of the pre-treatment period and the slope of the post-treatment period. The analysis compared the two period's slopes taking into account the treatment groups and each time point within a specific period. The actual parameter value at each time point was analyzed using a Mixed Model for Repeated Measures (MMRM) analysis (SAS®). The model included intercept and the time-point in continuous months (slope) as random effects and the following fixed effects and all their interactions: the period in the study as a class variable and the treatment group as a class. The changes from pre-treatment to post-treatment were explored by the estimated slopes resulting from the triple interaction of time point by period by group. An unstructured covariance structure was assumed and the denominator degrees of freedom were computed using the Kenward-Roger method. The change from baseline was analyzed using MMRM analysis (SAS®). The model included the fixed effects of the treatment group and scheduled visit as a categorical variable and their interaction. The model used the unstructured covariance matrix, the Restricted Maximum-Likelihood (REML) estimation method, and the Kenward-Roger adjustment method for the degrees of freedom.

## Results

Twenty-four patients were screened in this clinical study. Six patients failed the screening, mostly because they did not meet the minimum respiratory criterion of predicated SVC ≥ 70% or at least 10/12 in the ALSFRS-R respiratory sub-score. Additional 2 patients were screened but not enrolled, due to study discontinuation following the COVID-19 pandemic outbreak. Five patients enrolled in Group A (a single administration of 100 × 10^6^ AstroRx® cells). Four patients completed the 6-month follow-up under protocol Astro-001-IL, and 3 of them continued to the extension study Astro-002-IL and completed the entire 12-month follow-up after treatment. Eight patients enrolled in Group B (a single administration of 250 × 10^6^ AstroRx® cells) and 5 patients were treated. All 5 patients completed the 6-month follow-up and continued to the extension study, and 3 of them completed the entire 12-month follow-up. Three patients enrolled in Group C (2 administrations of 250 × 10^6^ AstroRx® cells). However, due to the COVID-19 outbreak, it was decided to discontinue the study for Group C, and no patient was treated in this group (Fig. 1b).

Before its injection, each formulated AstroRx® cell product was tested for the number of cells, viability, sterility profile, astrocytic cell identity, impurities, and potency to ensure release criteria defined for clinical batches are met (Table 1).

The baseline characteristics of the patients are presented in Table 2. Nine of the 10 treated patients of Group A and Group B were male, and all patients were white. All patients were stable on riluzole and none was treated with edaravone. The average age of the patients was 63 ± 4.9 in Group A and 61 ± 6.2 in Group B. All 5 patients in Group A had a diagnosis of probable ALS by El Escorial Criteria. In Group B, 3 patients had a diagnosis of probable ALS and 2 patients had a diagnosis of definite ALS. Nine of the 10 patients reported limb-onset of disease and 1 patient (Group B) reported a bulbar onset. The time from diagnosis was 14.5 ± 4.6 and 10.6 ± 2.0 months for Group A and Group B, respectively.

**Table 1:** Cell information

Part 1: Cell number, Viability and Safety profile of formulated AstroRx cell product								
Cohort	Patient Number	Cell count (X10^6^)	Cell viability (%)	Safety Profile				
				Bacteriology	Mycoplasma	Endotoxin level (EU/ml)	EPCAM (%)	SSEA4 (%)
A	1001	102	93.9	NC	NC	< 1.0	0.0	0.0
	1002	90	95.5	NC	NC	< 1.0	0.0	0.0
	1005	95	92.9	NC	NC	< 1.0	0.0	0.0
	1008	102	91.9	NC	NC	< 1.0	0.0	0.0
	1009	98.5	94.7	NC	NC	< 1.0	0.0	0.0
B	2010	245	96.6	NC	NC	< 1.0	0.0	0.0
	2012	270	97.6	NC	NC	< 1.0	0.0	0.0
	2015	258	96.6	NC	NC	< 1.0	0.0	0.0
	2016	261	96.4	NC	NC	< 1.0	0.0	0.0
	2017	265	95.6	NC	NC	< 1.0	0.0	0.0

Part 2: Cell characteristics of formulated AstroRx® product				
Cell characteristics		Cohort (Average ± SEM)		Release Criteria
		A	B	
Astrocytic identity	CD44 (%)	99.3 ± 0.2	99.8 ± 0.0	≥ 85%
	GLAST (%)	88.8 ± 7.6	69.9 ± 1.3	≥ 50%
	GFAP (%)	97.5 ± 0.9	92.2 ± 1.3	≥ 70%
Potency	TIMP-1 (ng/10^6^)	38.2 ± 7.7	43.9 ± 6.1	≥ 5 ng/106
	MIDKINE (ng/10^6^)	14.3 ± 1.3	19.9 ± 2.0	≥ 0.5 ng/106

*NC* no contamination, *EU* endotoxin unit, *EPCAM* epithelial cell adhesion molecule, *SSEA-4* stage-specific embryonic antigen-4, *CD44* cluster of differentiation 44, *GLAST* glutamate aspartate transporter, *GFAP* Glial fibrillary acidic protein.

Table I: Cell Characteristics of formulated AstroRx cell product. Part 1: Cell number, viability, and safety profile of AstroRx® cells used for intrathecal injection for each ALS patient. Part 2: AstroRx cell characteristics used for each study cohort.

**Table 2:** Patients’ Baseline Demographics

Characteristic	A (*n* = 5)	B (*n* = 5)
Gender		
Male	5 (100%)	4 (80%)
Female	0	1 (20%)
Race		
White	5 (100%)	5 (100%)
Age (years)	63 (4.9)	61 (6.2)
Height (cm)	173.4 (6.0)	170.6 (11.7)
Weight (kg)	65.2 (15.4)	72.8 (8.1)
BMI (m^2^/kg	21.6 (4.3)	25.2 (4.3)
ALS Diagnostic Criteria (revised El-Escorial)		
Definite	0.0	2 (40%)
Probable	5 (100%)	3 (60%)
Time from Diagnosis (months)	14.5 (4.6)	10.6 (2.0)
Riluzole Use	5 (100%)	5 (100%)
Initial Symptom		
Bulbar Onset	0	1 (20%)
Limb Onset	5 (100%)	4 (80%)
ALSFRS-R	35.6 (3.7)	34.2 (6.98)
% Predicted SVC	77.9 (14.2)	67.8 (18.9)
HHD Mega Score	− 1.36 (0.42)	− 0.52 (1.42)

Data are n (%) or mean (SD)

### Safety

Nine out of 10 (90%) of treated patients completed the 6-month follow-up, and 6 patients (60%) completed the 12-month follow-up. One patient in Group A and 2 patients in Group B died during the study, between 9 to 10 months post-treatment, due to respiratory failure that was associated with the natural progression of ALS. Table 3 summarizes the treatment-emergent adverse events (TEAE) reported in the study. All patients reported a total of 86 treatment-emergent adverse events (TEAE). None of TEAE was deemed to be associated with AstroRx® itself. Sixty-three TEAEs were mild, 19 were moderate, and 4 were severe. Six patients developed a total of 9 serious TEAE after the treatment, 2 patients in Group A and 4 patients in Group B (Additional file 2: Table S1). The most common TEAEs that were reported by at least 20% of the patients from Group A and Group B are shown in Additional file 2: Table S2. The Most frequent TEAE was post lumbar puncture (LP) headache, associated with IT injection procedure of the cells, and reported by 50% of the patients. Additional procedure-related TEAEs included pain in the injection site (30%), arthralgia, back pain, muscle contraction, and pain in the leg, each reported by 10% of the patients (Additional file 2: Table S3). All procedure-related AEs were graded as mild to moderate, and all were resolved. One event of moderate post-LP headache was resolved following a blood patch procedure that required hospitalization and was classified as an SAE. There was no apparent difference in the frequency or the nature of the procedure-related AEs between treatment groups. Three patients reported 4 AEs were related to mycophenolate mofetil, including headache, nausea, anemia, and hyperhidrosis (Additional file 2: Table S4). All the immunosuppression-related AEs were graded as mild to moderate, and all were resolved. No clinically significant changes were observed throughout the study in laboratory assessments, as well as in vital signs, physical examinations, or ECG results. MRI scans of the brain and spinal cord performed 6 months after AstroRx® cell injection showed no tumor formation in the CNS. Results were similar also after 12 months of follow-up, however, the MRI data at 12 months were very limited due to the inability of patients to perform MRI because of their medical condition, and restrictions imposed by the COVID-19 pandemic.

**Table 3:** Summary of TEAEs

Category	A		B		A + B	
	(N = 5)		(N = 5)		(N = 10)	
	Patients *n* (%)	Events *n*	Patients *n* (%)	Events *n*	Patients *n* (%)	Events *n*
Any TEAE	5 (100)	54	5 (100)	32	10 (100)	86
Death	1 (20)		2 (40)		3 (30)	
Any Serious TEAE	2 (40)	5	4 (40)	4	6 (60)	9
Any Severe TEAE	1 (20)	2	2 (40)	2	3 (10)	4
Any TEAE related to the study drug AstroRx®	0	0	0	0	0	0
Any TEAE related to IT Procedure	3 (60)	6	4 (80)	6	7 (70)	12
Any TEAE related to immunosuppression	1 (20)	2	2 (40)	2	3 (30)	4
TEAEs Severity						
Mild	5 (100)	41	5 (100)	22	10 (100)	63
Moderate	4 (80)	11	4 (80)	8	8 (80)	19
Severe	1 (20)	2	2 (40)	2	3 (10)	4

### Efficacy

#### ALSFRS-R

**Fig. 2:**
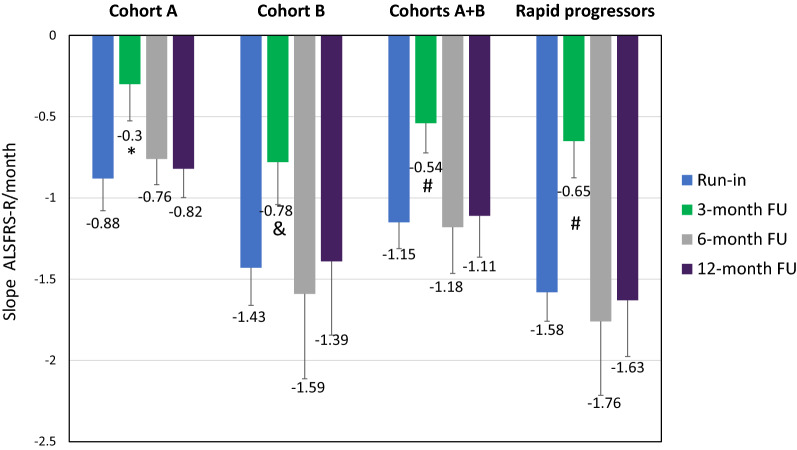
ALSFRS-R slopes analysis in run-in, and 3-, 6- and 12-month follow up after AstroRx® treatment. The change in slopes between pre-treatment slope ("Run-in") and post-treatment slope over 12 months was analyzed by using a repeated mixed model with fit least squares (LS) means (MMRM analysis). Analysis was performed on Cohort A, Cohort B, Cohort A&B as well as on rapid progressors (defined by ALSFRS-R≤1.1/month during run-in). * = P value=0.039 (Run-in vs. 3-month FU, & = P value=0.002 (Run-in vs. 3-month FU) and # = P value <0.001 (Run-in vs. 3-month FU)

The main outcome efficacy measure in the study was ALSFRS-R. At baseline visit (1 day before treatment) the mean ALSFRS-R score was 35.6 ± 3.7, 34.2 ± 7.0, 34.9 ± 5.3, and 33.4 ± 6.4 for Group A, Group B, combined Group A + B, and Rapid Progressors, respectively. The mean decline in the ALSFRS-R slope for patients in Group A was − 0.88/month during the run-in (3–4 months before treatment). In the first 3 months after AstroRx® cell injection, the mean decline of the ALSFRS-R slope was attenuated to − 0.3/month (p = 0.039), reflecting an attenuation of 66% in ALSFRS-R deterioration (Fig. 2). At 6 and 12 months after treatment, the ALSFRS-R deterioration rate was − 0.76/month and − 0.82/month, respectively-similar to that observed during run-in (Fig. 2). The mean deterioration of ALSFRS-R slope in Group B (− 1.43/month) during the run-in was greater than Group A (− 0.88/month). Similar to Group A, the ALSFRS-R deterioration rate during the first 3 months after treatment decreased to − 0.78/month (p = 0.002), representing an attenuation of 45% in ALSFRS-R decline. As observed in Group A, the attenuation of ALSFRS-R decline over the first 3 months post-treatment was not maintained at 6 and 12 months post-treatment (− 1.59/month and − 1.39/month, respectively) (Fig. 2). Combining the data of both groups demonstrated an attenuation of 53% in ALSFRS-R over the first 3 months post AstroRx® IT injection (p < 0.001), which was not maintained at 6- and 12-month follow-up (Fig. 2). The change in the ALSFRS-R slope was also analyzed in a subpopulation of rapid progressors from both groups (*n* = 5). Rapid progressors were defined as patients who deteriorated ≥ 1.1 ALSFRS-R points per month during the run-in period [25, 26]. The mean improvement in ALSFRS-R slope between the run-in period and 3-month follow-up in these patients was 58% (− 1.58/month vs. − 0.65/month, p < 0.001). Also in this subpopulation, after 3 months post single dosing the ALSFRS-R slope returned to a similar rate that was recorded before treatment (Fig. 2). An improvement ≥ 25% in the ALSFRS-R slope is considered clinically meaningful [27]. The individual ALSFRS-R slopes (Additional file 2: Figure S1) demonstrated an improvement of at least 25% in ALSFRS-R slope between the run-in and 3-month follow-up in 80% of the patients (4 patients in each group, data not shown).

#### Hand-held dynamometry (HHD)

A comparison of the HHD megascore slope between run-in and 3-month follow-up showed a trend of improvement in both Group A and Group B, which was not statistically significant (Additional file 2: Table S5). In combined Group A + B, the rate of HHD megascore decline in the run-in period was − 0.06 ± 0.028 vs. − 0.02 ± 0.031 during the first 3 months post-treatment (p = 0.24) and in Rapid Progressors − 0.06 ± 0.053 vs. + 0.01 ± 0.060. At 6- and 12-month follow-ups, the HHD megascore returned to the similar decline rate that was recorded in the run-in period.

### Slow vital capacity

At the baseline visit, the mean percent of predicted SVC (%SVC) was 77.9 ± 14.2%, 67.8 ± 18.9%, 72.9 ± 16.6%, and 66.6 ± 17.1% for Group A, B, combined A + B, and Rapid Progressors, respectively. A comparison of %SVC rate deterioration between the run-in period and follow-up showed a continuation of %SVC deterioration in both Group A and Group B, and Rapid Progressors (Additional file 2: Table S6). The rate of decline in %SVC rate in combined Group A + B (n = 10) was –1.08 ± 1.04% in run-in vs. − 3.20 ± 1.10% during the first 3 months of follow-up (p = 0.01), and − 3.09% ± 0.59% during the 12- month follow-up (p = 0.01).

### Serum neurofilament light chain (Nfl)

Serum samples for the analysis of Nfl as a biomarker for ALS disease progression were collected throughout the study, before and after treatment. Nfl, which was extensively studied in ALS, is proposed as a potential biomarker for ALS diagnosis and prognosis [28, 29]. The proposed cut-off serum Nfl level to differentially distinguish between ALS patients vs. non-neurodegenerative controls is 49 pg/mL [30, 31]. The serum Nfl concentration in six of the patients was greater than 49 pg/ml throughout the study and levels tended to be higher in rapid progressors, as reported by others [32] (data not shown). However, no clear tendency of change in the kinetics of serum Nfl was observed (Additional file 2: Figure S2).

## Discussion

Although the pathogenesis of MN death in ALS is not fully elucidated, malfunctioning astrocytes can contribute to the death of MNs and the progression of the disease. A cell therapy approach that includes intrathecal injection of healthy and functional astrocytes may compensate for the diseased endogenous astrocytes and attenuate the disease progression. AstroRx® cell therapy is composed of healthy astrocytes derived from human embryonic stem cells. Intrathecal injection of AstroRx allows the distribution of the cells throughout the neural axis, where it can affect both upper and lower MNs [19]. Moreover, AstroRx® is an allogeneic “off-the-shelf” product that does not require individual production procedures as in autologous cell therapies.

This first-in-human phase I/IIa clinical trial assessed the safety and preliminary efficacy of a single intrathecal injection of AstroRx® in two doses. Ten patients were enrolled in the study, 5 in each treatment dose. No AEs related to the product itself were reported. The most common AEs were related to the intrathecal administration procedure or treatment with MMF. These AEs were mild to moderate, and all resolved either spontaneously or with treatment.

Reported SAEs were related to the expected progression of ALS. Three patients died due to the natural progression of ALS 9 to 10 months post-treatment.

A potential major safety concern in using embryonic stem cells as a source for cell therapy is their potential to form teratomas. Before its intrathecal cell injection, each AstroRx® cell product was tested to meet the acceptance criteria for pluripotent markers. MRI scans of the spinal cord and brain performed 6 months after cell injection did not reveal any tumor or teratoma formation. Overall, these safety data indicate that a single injection of AstroRx® at both tested doses of 100 × 10^6^ and 250 × 10^6^ is safe and well-tolerated.

The patient population enrolled in this study was at a relatively early disease stage (about 18 months from first symptoms) and the ALSFRS-R at baseline was similar between groups. The percentage of male patients in the study was 90%, profoundly greater than the estimated ALS incidence ratio of 1.29:1 between males and females [33], which may be explained by the small number of subjects included in this study. Although the known mechanisms of action of AstroRx® are assumed to influence both genders similarly, in future larger clinical studies, efforts will be made to include patients that better reflects the gender ratio in the ALS population. The disease progression recorded during the run-in period was on average greater in patients of Group B. The progression of ALS was assessed by pre-post analysis of slope analysis, change from baseline, and responder analysis. The analyses were performed also on a subpopulation of rapid progressors (ALSFRS-R ≥ 1.1/month during the run-in period). A clinically meaningful signal of decline in disease progression, as assessed by the ALSFRS-R score, was observed for the first 3 months after treatment, as compared to the pre-treatment period. Although the deterioration in Group B patients was greater than that of Group A, the trend of effect was similar. A similar trend was also observed in the rapid progressor population suggesting that AstroRx® has the potential to be effective in a broader ALS patient population. The additional outcome measures of Muscle strength as measured by HHD showed a trend of slowdown in deterioration for the first 3 months post-treatment as compared to the run-in period but was not statistically significant. In contrast, respiratory function expressed as predicated %SVC continued to deteriorate during the entire follow-up, including the first 3 months post AstroRx® injection. No clear trend of change was observed in serum marker Nfl between the pre- and post-treatment periods.

The interpretation of the efficacy results is limited by the small sample size and the difference in disease progression before treatment between study groups. Yet the trend of attenuation in disease progression for the first 3 months as reflected by ALSFRS-R was observed in 8 out of 10 patients from both groups. Notably, in larger studies supporting FDA market authorization, edaravone and the recently ALS-approved drug and Relivrio™, showed a modest but statistically significant benefit in slowing down ALSFRS-R decline, although they did not demonstrate a significant improvement in other ALS outcome measures [6, 34]. The effect of AstroRx® on ALSFRS-R, as well as the other ALS outcome measures, should be further evaluated in a larger randomized parallel, placebo-controlled clinical trial.

The duration of the effect of AstroRx® may be related to the survival of the cells in the CNS. In a preclinical study in immunodeficient mice, AstroRx® cells were shown to survive in the CNS at the pre-specified endpoint of the study 9 months after the intrathecal injection [19]. Although the CNS is generally considered an immune-privileged site, foreign antigens can still drain from the CNS to the peripheral lymph nodes through the glymphatic system and may trigger an immune reaction [35, 36]. AstroRx® is composed of allogeneic cells which can potentially elicit such an immune attack following their injection into the CSF. Clinical trials involving allogeneic cell transplantation in the CNS implement a single or combined immunosuppression regimen to avoid graft rejection [37–39]. In our study, transient mild immunosuppression by oral mycophenolate mofetil for one month following AstroRx® intrathecal injection was applied. Currently, there is no evidence from other clinical trials that immunosuppression changes the course of ALS disease [40–42]. Therefore we assume that the therapeutic benefit observed during the first 3 months following AstroRx® is not related to the concomitant immunosuppression over the first month post AstroRx® administration.

The survival of the cells in the CNS of the patient was not investigated in this clinical study. It cannot be excluded that the reduction in the clinical signal of effect after 3 months is a result of the loss of AstroRx® cells. Nevertheless, there is no indication for a systemic immune response following intrathecal AstroRx® cell injection as measured by blood immunoglobulins before and after treatment, or change in ɣ-interferon release by peripheral blood mononuclear cells (PBMC) collected from the treated patient before and after treatment (data not shown). The survival of AstroRx® cells post intrathecal cell injection and the regimen of immunosuppression will further be explored in the next clinical study.

## Study limitations

The interpretation of the study results is limited by its exploratory nature and small study sample size. The study was unblinded with no control arm, making it difficult to estimate the net effect of the treatment. In addition, there was an imbalance in the pre-treatment ALSFRS-R deterioration rate between the 2 study cohorts (Cohort A − 0.88/month and B − 1.43/month), which makes it difficult to determine whether there is a difference in the effectiveness of the 2 tested doses. The pre-post analysis of the efficacy outcome measures assumes linearity in the deterioration. However, while ALS clinical trials assume linear deterioration in ALSFRS-R over time [6, 34], there is also evidence suggesting that ALS may progress in a non-linear fashion [43, 44]. This study did not assess transplant engraftment; therefore we have no data about the survival of AstroRx® in patients and the effectiveness of immunosuppression to prevent rejection.

## Conclusions

In conclusion, a single IT administration of AstroRx®, an astrocyte cell-based therapy derived from embryonic stem cells, at a dose of 100 × 10^6^ or 250 × 10^6^ cells is considered safe. A signal of beneficial clinical effect was observed over the first 3 months post single treatment. It remains to be investigated whether repeated IT administrations of AstroRx® may prolong its beneficial effect in ALS. To further determine the clinical effect of AstroRx in ALS, additional powered, controlled clinical studies to evaluate repeated administration of AstroRx, e.g. every 3 months, are required.

## Supplementary Information

**Additional file 1.** Supplementary materials and methods.

**Additional file 2: Figure S1.** ALSFRS-R slope for each patient in run-in, and 3-, 6- and 12-month follow up after AstroRx® treatment. **Figure S2.** Serum levels of Nfl for each patient. **Table S1.** List of serious TEAEs. **Table S2.** Treatment emergent adverse events (TEAE) reported in at least 20% of patients in both treatment arms. **Table S3.** AEs related to IT injection of AstroRx cells by lumbar puncture. **Table S4.** AEs related to immunosuppression by Mycophenolate Mofetil. **Table S5.** Slope analysis of handheld dynamometer megascore. **Table S6.** %SVC slopes analysis in run-in, and 3-, 6- and 12-month follow up after AstroRx® treatment

## Data Availability

The datasets used and/or analyzed during the current study are available from the corresponding author on reasonable request.

## Abbreviations

AE: Adverse event
ALS: Amyotrophic lateral sclerosis
ALSFRS-R: ALS Functional Rating Scale-Revised
ALSAQ-40: ALS Assessment Questionnaire
APC: Astrocyte progenitor cells
AQP-4: Aquaporin-4
BCV: Blood count visit
BDNF: Brain-derived neurotrophic factor
b.i.d.: Twice a day
BMI: Body mass index
BW: Body weight
CBC: Complete blood count
CK: Creatine kinase
CNS: Central nervous system
CNTF: Ciliary neurotrophic factor
Cr: Creatinine
CSF: Cerebrospinal fluid
C-SSRS: Columbia-Suicide Severity Rating Scale
CT: Computed tomography
CXCR: C-X-C chemokine receptor
DSMB: Data safety monitoring board
eCRF: Electronic case report form
EC: Ethics committee
ELISA: Enzyme-linked immunosorbent assay
FU: Follow-up
GDNF: Glial derived neurotrophic factor
GFAP: Glial fibrillary acidic protein
GLAST: Glutamate aspartate transporter
hESC: Human embryonic stem cells
HHD: Hand-held dynamometer
ITT: Intention to treat
IT: Intrathecal
LP: Lumbar puncture
MedDRA: Medical Dictionary for Regulatory Activities
mITT: Modified Intent to Treat
MMF: Mycophenolate mofetil
MMRM: Mixed-effect model for repeated measures
MN: Motor neuron
MoH: Ministry of Health
MRI: Magnetic resonance imaging
Nfl: Neurofilament light chain
OCT-4: Octamer-binding transcription factor-4
pNFH: Phosphorylated neurofilament heavy chain
PBMC: Peripheral blood mononuclear cells
SAE: Serious adverse event
SD: Standard deviation
SEM: Standard error of the mean
SSEA: Stage-specific embryonic antigen
SVC: Slow vital capacity
