# Supplementary material for: Safety and efficacy of first-in-man intrathecal injection of human astrocytes (AstroRx®) in ALS patients: phase I/IIa clinical trial results
Source: J Transl Med. 2023 Feb 14;21:122. doi: 10.1186/s12967-023-03903-3 (PMC9927047; doi:10.1186/s12967-023-03903-3)
Supplement: Supplementary file 1 — Additional file 1. Supplementary materials and methods. [file 12967_2023_3903_MOESM1_ESM.docx]

**Supplementary Materials and Methods**

**Inclusion and exclusion criteria for study Astro-001**

**Inclusion Criteria**

Prior to enrolment into this study, patients must meet ALL of the following inclusion criteria:

1. Patient with sporadic or familial ALS diagnosed as probable or definite ALS patients according to the World Federation of Neurology El Escorial criteria (revised) (APPENDIX 2).

2. Males and females between 18 and 70 years of age.

3. Patients with an ALS-FRS-R score of ≥30 at Screening Visit and with an ALS diagnosis of two years or less

4. No history of active psychiatric disorder. Patients receiving antidepressants as a preventive treatment, with no history of active psychiatric disorder may be included.

5. Patient has a good understanding of the study and nature of the procedure and is expected to be able to comply with visit schedules and assessments without difficulty.

6. Patient provides written informed consent prior to any study procedure.

7. Females of childbearing potential must have a negative serum pregnancy test and must use two medically acceptable methods of contraception during the course of the study, not including the rhythm method. Effective birth control measures include hormonal contraception, a barrier method such as a diaphragm, intrauterine device (IUD) and/or condom with spermicide or abstention from sexual intercourse. A female who is surgically sterile and/or postmenopausal (defined as at least 1 year without menses as demonstrated by medical history or patient report), is not considered to be of childbearing potential.

8. Male patients must use an effective method of contraception during the course of the study. These include condom, having undergone a vasectomy or abstain from sexual intercourse.

9. Patient is medically able to undergo an intrathecal injection (IT) into the CSF as determined by the investigator, surgeon or anaesthesiologist

10. Patient is up-to date with influenza vaccination if enrolled during the influenza seasonal vaccination program; if enrolled outside the influenza season, the patient must agree to be vaccinated at the beginning of the vaccination season.

11. Patients should either be on a stable dose of Riluzole and/or Edaravone) Radicava®) (if applicable) for at least 30 days, or not be treated with Riluzole and/or Edaravone (usually because of previous adverse effects or a decision by the patient). Patients not treated with Riluzole and/or Edaravone will not start Riluzole and/or Edaravone treatment during the study period.

12. Patient is medically able to tolerate immunosuppression regimen consisting of Mycophenolate Mofetil at 1gr b.i.d. for 1 month, starting 2 days before transplantation as determined by the investigator

13. Presence of a willing and able caregiver who understands the need to attend all follow-up visits, even if mobility declines.

**Exclusion Criteria**

Patients will be excluded from the study if ANY of the following conditions are present:

1. Patient has a past infection or a positive test for HBV, HCV or HIV.

2. Patient is in need of respiratory support.

3. Patient has a lower than 10/12 in ALS-FRS-R respiratory parameters or below 70% of predicted SVC.

4. Patient has renal failure (eGFR <30 mL/min according to Cockcroft formula).

5. Patient has impaired hepatic function (Alanine Transaminase (ALT), Aspartate aminotransferase (AST) or Gamma-Glutamyl Transferase (GGT) 2-fold higher than normal upper limit).

6. Patient has a Body Mass Index (BMI) of <18.5 or > 30.

7. Patient suffers from significant cardiac disease, diabetes, autoimmune diseases, chronic severe infection, malignant disease or any other disease or condition that may risk the patient or interfere with the ability to interpret the study results

8. Patient has systemic inflammation or active infections.

9. Patient has been treated previously with any stem cell therapy.

10. Current use of immunosuppressant medication or use of such medication within 6 weeks of Screening visit (Visit 0)

11. Patient has participated in another clinical treatment trial or received other experimental medications outside of a clinical trial within 1 month prior to start of this study.

12. Patient has any of the following:

a. Hemoglobin < 10 g/dL (G%)

b. Total WBC < 4000 (4X103/µL)

c. Uncontrolled diabetes (defined as hemoglobin A1c >8%)

d. Known active GI bleeding

13. Any known immunodeficiency syndrome.

14. Any concomitant disease or condition limiting patient safety to participate including:

a) Coagulopathy

b) Hypotension requiring vasopressor therapy

c) Previous spinal surgery at the site of planned transplantation

d) Skin breakdown over the site of LP

e) Active malignancy (except for non-melanoma skin cancer)

f) Spinal stenosis

g) Uncontrolled hypertension (defined as systolic >180 or diastolic >100)

h) Current drug abuse or alcoholism

i) Significant cognitive impairment, clinical dementia, or psychiatric illness

j) Neurodegenerative disease e.g. Parkinson’s disease, Alzheimer’s disease

k) Suicidality rating (Columbia-Suicide Severity Rating Scale - C-SSRS) equal to levels 6-10) i.e., has exhibited any suicidal behaviours

l) Significant pulmonary disorder not attributed to ALS or requires treatment that might complicate the evaluation of ALS on respiratory function

m) Known hypersensitivity to mycophenolate mofetil such as suffering from phenylketonuria (PKU)

**Calculation of HHD megascore**

The limb strength measured by the Handheld Dynamometer (HHD) was measured in KG/F and for the following organs (left and right sides):

1. shoulder flexion
2. elbow flexion
3. elbow extension
4. wrist extension
5. first dorsal interosseous
6. hip flexion
7. knee extension
8. knee flexion
9. ankle dorsiflexion

The HDD mega-score was calculated by averaging the standardized 18 organs scores (9 organs, left and right sides).

The standardized HDD megascore was calculated according to the following formulae for each patient and visit:

Standardized score = (Organ score – Mean)/SD;

Where Mean and SD for each organ are derived from: Shefner JM, Liu D, Leitner ML, et al. Quantitative strength testing in ALS clinical trials. Neurology. 2016;87(6):617-624.
