# Supplementary material for: Safety and efficacy of first-in-man intrathecal injection of human astrocytes (AstroRx®) in ALS patients: phase I/IIa clinical trial results
Source: J Transl Med. 2023 Feb 14;21:122. doi: 10.1186/s12967-023-03903-3 (PMC9927047; doi:10.1186/s12967-023-03903-3)
Supplement: Supplementary file 2 — Additional file 2: Figure S1. ALSFRS-R slope for each patient in run-in, and 3-, 6- and 12-month follow up after AstroRx® treatment. Figure S2. Serum levels of Nfl for each patient. Table S1. List of serious TEAEs. Table S2. Treatment emergent adverse events (TEAE) reported in at least 20% of patients in both treatment arms. Table S3. AEs related to IT injection of AstroRx cells by lumbar puncture. Table S4. AEs related to immunosuppression by Mycophenolate Mofetil. Table S5. Slope analysis of handheld dynamometer megascore. Table S6. %SVC slopes analysis in run-in, and 3-, 6- and 12-month follow up after AstroRx® treatment [file 12967_2023_3903_MOESM2_ESM.docx]

**eFigure 1: ALSFRS-R slope for each patient in run-in, and 3-, 6- and 12-month follow up after AstroRx® treatment**


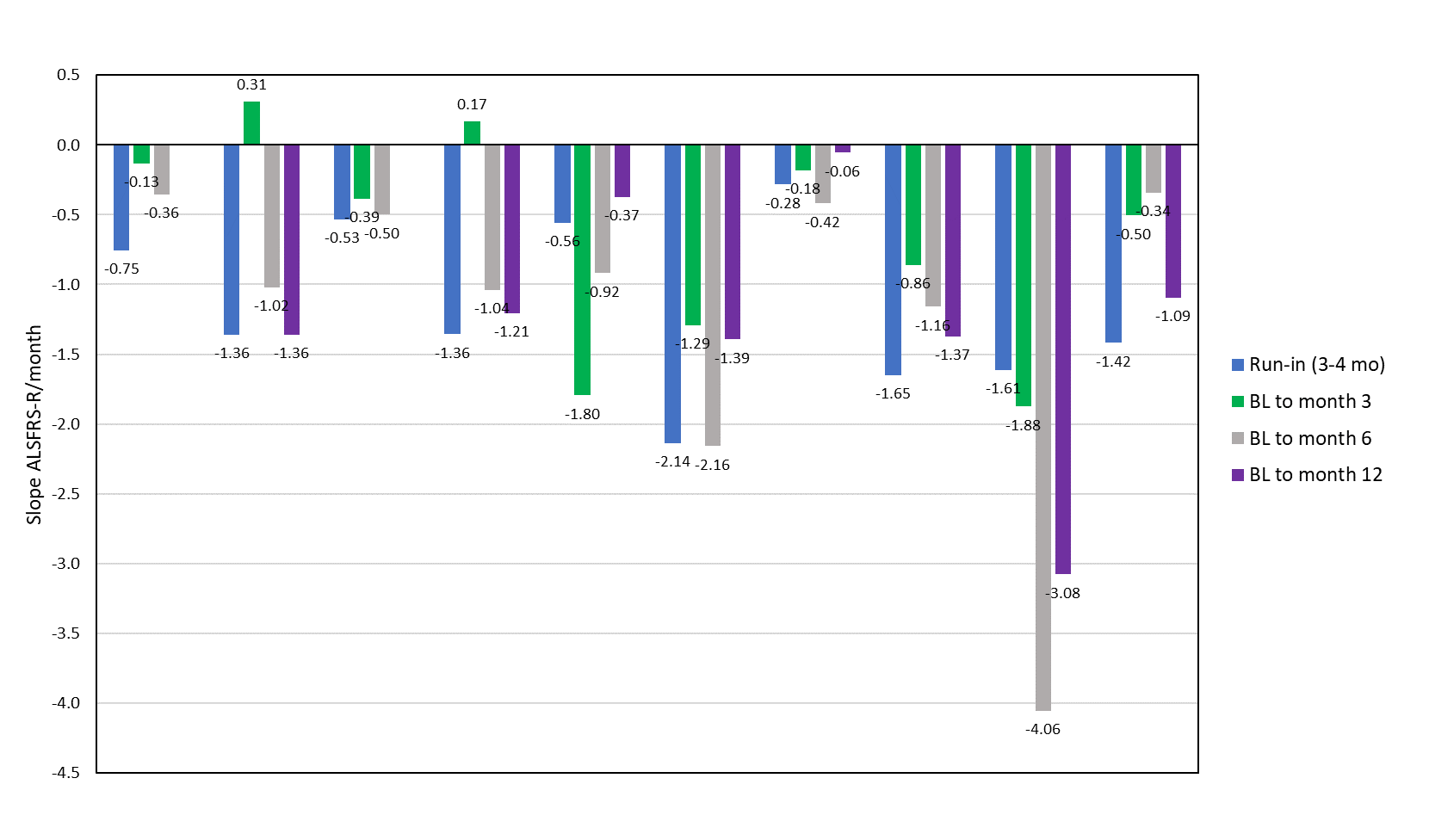


1002/A

1005/A

1008/A

1001/A

1009/A

2010/B

2012/B

2015/B

2016/B

2017/B

(1 mo)

(9 mo)

(10 mo)

**eFigure 2: Serum levels of Nfl** ­­for each patient

*
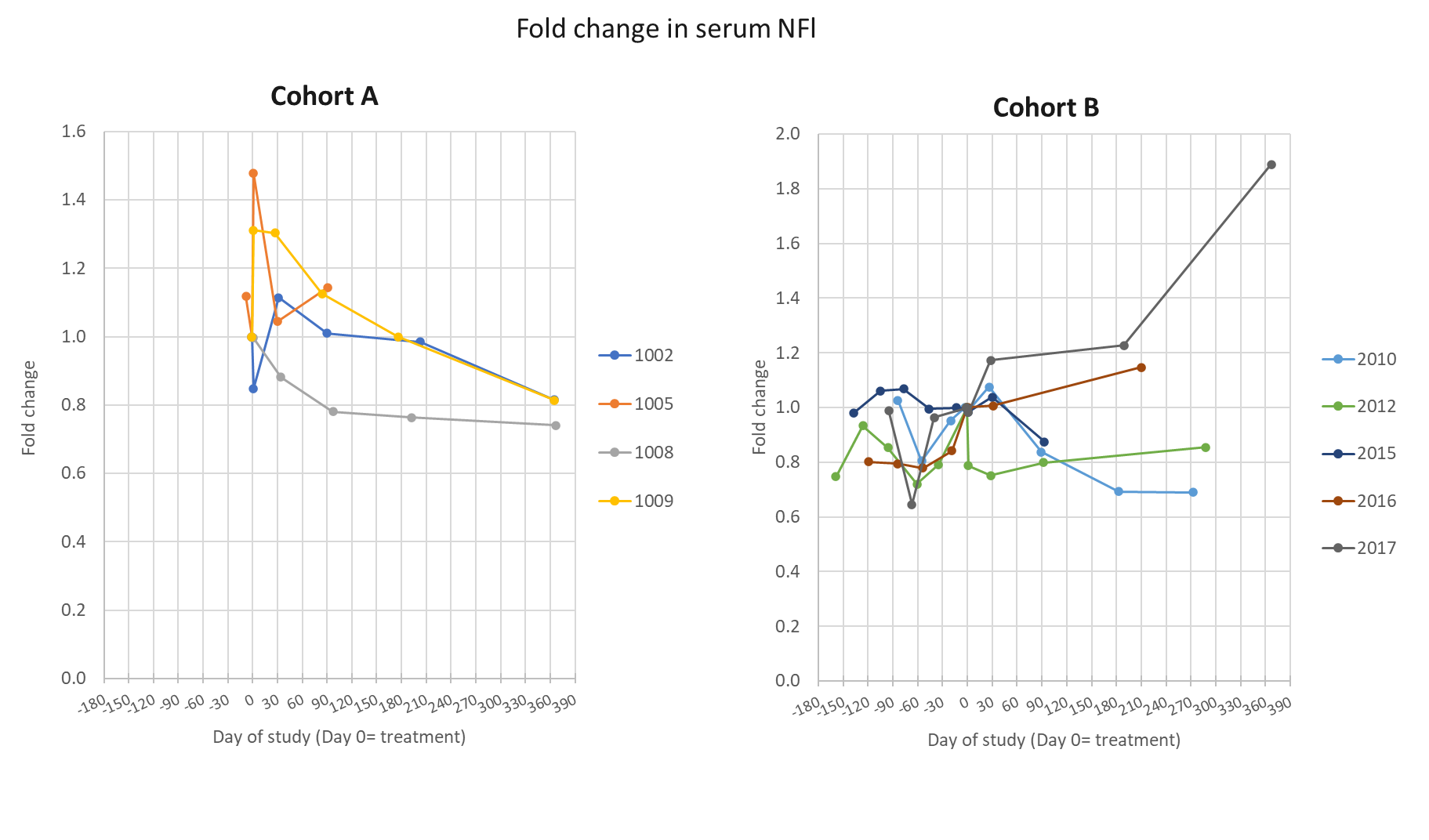
*

**eTable 1: List of serious TEAEs**

| Patient ID/  cohort | AE preferred term | SAE start day (days after treatment) | SAE end day (days after treatment) | Relation to  drug product/  IT procedure/  immunosuppression | SAE outcome |  |
| --- | --- | --- | --- | --- | --- | --- |
|  |  |  |  |  |  |  |
| 1005/A | Gastrostomy | 70 | 71 | Not related | resolved |  |
|  | Pneumonia aspiration | 165 | 178 | Not related | resolved |  |
|  | Viral infection | 198 | 202 | Not related | resolved |  |
|  | Respiratory failure | 274 | Death | Not related | Death |  |
| 1008/A | Dysphagia | 153 | 155 | Not related | resolved |  |
| 2012/B | Procedural headache | 0 | 17 | Related to IT procedure | resolved |  |
| 2015/B | Respiratory failure | 289 | Death | Not related | Death |  |
| 2016/B | Respiratory failure | 303 | Death | Not related | Death |  |
| 2017/B | Cerebellar infarction | 188 | Ongoing | Not related | No clinical signs related to the SAE were reported |  |

**eTable 2: Treatment emergent adverse events (TEAE) reported in at least 20% of patients in both treatment arms**

| Preferred Term | A | | B | |
| --- | --- | --- | --- | --- |
|  | (n=5) | | (n=5) | |
|  | Patients n (%) | Events n | Patients n (%) | Events n |
| Procedural Headache | 3 (60) | 3 | 2 (40) | 2 |
| Oedema Peripheral | 3 (60) | 4 | 0 | 0 |
| Respiratory failure | 1 (20) | 1 | 2 (40) | 2 |
| Injection Site Pain | 1 (20) | 1 | 2 (40) | 2 |
| Dyspnoea | 0 | 0 | 2 (40) | 3 |
| Anaemia | 1 (20) | 1 | 1 (20) | 1 |
| Nausea | 0 | 0 | 2 (40) | 2 |
| Bronchitis | 1 (20) | 1 | 1 (20) | 1 |
| Neck Pain | 2 (40) | 2 | 0 | 0 |
| Ecchymosis | 2 (40) | 2 | 0 | 0 |

| Preferred Term | Cohort A  (*n*=5) | | Cohort B  (*n*=5) | | Cohort A+B  (*n*=10) | |
| --- | --- | --- | --- | --- | --- | --- |
|  | Patients  *n* (%) | Events  *n* | Patients  *n* (%) | Events  *n* | Patients  *n* (%) | Events  *n* |
| Procedural headache | 3 (80) | 3 | 2 (40) | 2 | 5 (50) | 5 |
| Injection site pain | 1 (20) | 1 | 2 (40) | 2 | 3 (30) | 3 |
| Arthralgia | 1 (20) | 1 | 0 | 0 | 1 (10) | 1 |
| Back pain | 1 (20) | 1 | 0 | 0 | 1 (10) | 1 |
| Muscle contractions involuntary | 0 | 0 | 1 (20) | 1 | 1 (10) | 1 |
| Pain in extremity | 0 | 0 | 1 (20) | 1 | 1 (10) | 1 |
| Total | 3 (60) | 6 | 4 (80) | 6 | 7 (70) | 12 |
| Severity | | | | | | |
| Mild | 2 (40) | 4 | 2 (40) | 2 | 5 (50) | 8 |
| Moderate | 3 (60) | 4 | 2 (40) | 2 | 4 (40) | 4 |
| Severe | 0 | 0 | 0 | 0 | 0 | 0 |

**eTable 3: AEs related to IT injection of AstroRx cells by lumbar puncture**

| Preferred Term | A | | B | | A+B | |  |
| --- | --- | --- | --- | --- | --- | --- | --- |
|  | (N=5) | | (N=5) | | (N=10) | |  |
|  | Patients n (%) | Events m | Patients n (%) | Events m | Patients n (%) | Events m |  |
|  |  |  |  |  |  |  |  |
| Headache | 1 (20) | 1 | 0 | 0 | 1 (10) | 1 |  |
| Nausea | 1 (20) | 1 | 0 | 0 | 1 (10) | 1 |  |
| Anemia | 0 | 0 | 1 (20) | 1 | 1 (10) | 1 |  |
| Hyperhidrosis | 0 | 0 | 1 (20) | 1 | 1 (10) | 1 |  |
| Total | 1 (20) | 2 | 2 (40) | 2 | 3 (30) | 4 |  |
| Severity | | | | | | |  |
| Mild | 0 | 0 | 2 (40) | 2 | 2 (40) | 2 |  |
| Moderate | 1 (20) | 2 | 0 | 0 | 1 (10) | 2 |  |
| Severe | 0 | 0 | 0 | 0 | 0 | 0 |  |

**eTable 4: AEs related to immunosuppression by Mycophenolate Mofetil**

**eTable 5: Slope analysis of handheld dynamometer megascore**

|  |  |  | **Cohort** | | | |
| --- | --- | --- | --- | --- | --- | --- |
|  |  | **Statistics** | **A (*n*=5)** | **B (*n*=5)** | **A+B (*n*=10)** | **A+B Rapid Progressors (*n*=5)** |
| **Pre-treatment** | **Run-in (3-4 months)** | Slope Estimate | -0.09 | -0.03 | -0.06 | -0.06 |
|  |  | SE | 0.022 | 0.051 | 0.028 | 0.053 |
| **Post-treatment** | **3 months** | Slope Estimate | -0.08 | 0.04 | -0.02 | 0.01 |
|  |  | SE | 0.024 | 0.059 | 0.031 | 0.060 |
|  |  | p-value | 0.5797 | 0.2555 | 0.2430 | 0.2045 |
|  | **6 months** | Slope Estimate | -0.08 | -0.08 | -0.08 | -0.10 |
|  |  | SE | 0.022 | 0.043 | 0.023 | 0.039 |
|  |  | p-value | 0.4537 | 0.4047 | 0.4740 | 0.4387 |
|  | **12 months** | Slope Estimate | -0.07 | -0.07 | -0.07 | -0.08 |
|  |  | SE | 0.023 | 0.031 | 0.019 | 0.033 |
|  |  | p-value | 0.1247 | 0.5005 | 0.7153 | 0.7562 |

**eTable 6: %SVC slopes analysis in run-in, and 3-, 6- and 12-month follow up after AstroRx® treatment**

|  |  |  | **Cohort** | | | |
| --- | --- | --- | --- | --- | --- | --- |
|  |  | **Statistics** | **A (n=5)** | **B (n=5)** | **A+B (n=10)** | **A+B Rapid Progressors (n=5)** |
| **Pre-treatment** | **Run-in (3-4 months)** | Slope Estimate (%) | -0.39 | -1.98 | -1.08 | -2.82 |
|  |  | SE | 0.765 | 1.955 | 1.014 | 1.442 |
| **Post-treatment** | **3 months** | Slope Estimate (%) | -2.86 | -3.75 | -3.2 | -3.61 |
|  |  | SE | 0.922 | 2.103 | 1.105 | 1.646 |
|  |  | p-value | 0.0277 | 0.2154 | 0.0136 | 0.5567 |
|  | **6 months** | Slope Estimate (%) | -3.03 | -4.60 | -3.82 | -4.48 |
|  |  | SE | 0.747 | 1.764 | 0.922 | 1.339 |
|  |  | p-value | <.0001 | 0.0098 | <.0001 | 0.0656 |
|  | **12 months** | Slope Estimate (%) | -2.49 | -3.57 | -3.09 | -3.36 |
|  |  | SE | 0.673 | 0.976 | 0.591 | 0.796 |
|  |  | p-value | 0.0015 | 0.4508 | 0.0104 | 0.9448 |
